# Texas State Health Board’s Campaign for the Eradication of Malaria

**Published:** 1916-11

**Authors:** 


### Texas State Health Board's Campaign for the Eradication of Malaria.

In the early part of 1915, the International Health Board, a department of the Rockefeller Foundation, amended its charter, which had covered only hookworm, and enlarged the scope and changed the plans so as to cover those diseases due to soil pollution along with an education campaign leading to the prevention of sickness under the title of Intensive Health Work.

This work was placed under the supervision of a State director for Texas. In each county where the work is done, a field director, who is a physician, is placed as head of the county work, and with a microscopist and two carpenters, compose the county force. It is a well known fact that in that part of Texas where hookworm and dysentery are prevalent that malaria is also prevalent. Since the International Health Board was not at that time ready to undertake the eradication of malaria, the Extension Department of the University of Texas made a malarial survey in conjunction with the other work that was being done. A microscopist for the malarial work was placed in each county by the University, and so far as the limited appropriation would go an attempt was made to eradicate malaria in those districts by recommending treatment of those infected and the proper screening of homes.

The statistics gathered in this survey, as well as the results of the attempt to eradicate the disease in this limited way, are intensely interesting and most gratifying, as shown by the action of the Leon County Medical Society. At the October meeting a report of the work was made. Drs. Seale and Carrington of Marquez reported thirteen calls to see malarial patients this summer, while last summer their records showed more than 400, in spite of the fact that malaria has been more prevalent in the summer of 1916 than in several years past. Dr. Boggs of Vanetia reported a decrease of more than 50 per cent, and it was agreed that these cases were in families that did not follow the advice given during the campaign.

The county society unanimously adopted the following resolution:

"Whereas, The four months' intensive health work in this county has been completed, and

"Whereas, The result has been so eminently successful, be it

"Resolved, That provision should be made for the continuation of this work until the entire county has been covered at least as well as the communities of Leona, Vanetia and Marquez.

(Signed) "DRS. ROSS AND SMITH,
"Committee.

"Motion was carried that Drs. Ross, Spruell and Powell be appointed as a committee to go before the commissioners court of this county next Tuesday (October 10, 1916), and urge the appropriation of funds necessary to continue the work in Leon county.

"Moved also that every member of the society be an ex-officio member of this committee and that as far as possible all be present at the meeting of the commissioners court."

In the beginning of the present work in Texas six counties were visited. Four of them agreed to make an appropriation of $800 for four months' work in three rural districts. The International Health Board appropriated a like amount, and at the same time the University bore an equal amount of the expense. In this way the county secured the services of expert men by appropriating only one-third of the cost. While the work of the International Health Board is not limited as to the number of counties or the amount of appropriation, the work done by the Extension Department of the University depends upon the action of the Board of Regents, and it is the wish of the State Health Department that the University Regents may see fit to make an appropriation sufficiently large to place this work on a permanent basis, for it is the belief that since the University is partly supported by the taxpayers, that the taxpayers are entitled to some education that will lead to the protection of their homes from the invasion of disease.

While the purpose of the work is the eradication of hookworm, malaria, and the control of soil pollution leading to the prevention of typhoid, dysentery, intestinal parasite and soil borne disease, the lines of endeavor in preventive medicine are so closely interlocked that this intensive health work covers the entire field.

At regular periods lectures, illustrated by the stereopticon and charts, are delivered on other epidemic diseases, as well as those above. In the house-to-house canvass that is made to secure statistics, personal advice is given and literature is distributed, relating to infant care, home and personal hygiene, as well as communicable diseases.

When a county has accepted the proposition and has made the appropriation of $800 for four months' work, or $2400 for the year, the State director visits the rural communities and delivers illustrated lectures at the rural schoolhouses. An agreement is circulated among the people to be signed by the heads of the families. When these agreements are returned, the three communities showing the highest percentage of signatures are selected. In Leon county three communities signed up to 100 per cent. In each of the three communities a laboratory is established and a microscopist is placed in charge. The field director, assisted by the microscopist, makes a sanitary survey of the district, and on a map locates every home, showing the number of inmates, the type of privy and all disease-breeding conditions. At the same time containers for specimens are left with the families to be returned for examination. Each person in the family is examined and directions for treatment are given.

When the survey is completed, the patients are treated for hookworm. The treatment for malaria and other diseases is under the care of the family physician with the assistance of the physicians in charge of the work. Two carpenters are employed, who go from house to house, and with the assistance of the householder, build sanitary privies. They also give directions for the proper screening of homes, draining or kerosening stagnant pools and the elimination of disease-producing conditions. When the work is completed every home and public building where the occupants will co-operate has been placed in a sanitary condition.

The statistics gathered in Leon county in reference to malaria show that the disease cost per capita more than $2.60 for drugs and doctor's bills. They lost nine days, or $5.00 per person, per year. One person out of 383 died from the disease. The cost of the campaign, $1.64 per capita, of which amount Leon county paid 54⅔ cents.

East of the 30-inch rainfall belt there are eighty-eight counties with an altitude of 500 feet or less. These counties are known as the malarial belt of East Texas. The rural population of these counties represent 44 per cent of the entire State's population. They are $5.70 per head poorer in personal property than the average for the State. They work 27 per cent of the State's farm land; they carry 37 per cent of the farm mortgages in the State; they pay 55 per cent of all taxes; they raise 65 per cent of all crops raised in the State.

If the statistics gathered in Leon county be applied to this malarial belt as a whole, it is interesting to note that these eighty-eight counties pay for malaria a doctor's and drug bill of more than $5,031,000; from loss of time due to this disease they lose more than $9,675,000. There are over 1,000,000 persons infected, and more than 5000 deaths per year.

The cost of a campaign such as decreased the disease more than 50 per cent in the three districts of Leon county would cost the State a little more than $100,000, and would add only 4 mills to the present tax rate of 55 cents. Is the game worth the candle? If East Texas is taxed to appropriate $100,000 for the destruction of jack rabbits and coyotes in West Texas, it is but fair to tax West Texas to appropriate $100,000 for Intensive Health Work resulting in the eradication of hookworm and the limitation of malaria, pellagra and typhoid fever, and the game is well worth the candle.

